# Exclusion and Inequality in Late Working Life–On the Gendered Risks for Old-Age Exclusion in Sweden and Poland

**DOI:** 10.1093/geroni/igaa057.211

**Published:** 2020-12-16

**Authors:** Andreas Motel-Klingebiel, Jolanta Perek-Białas, Indre Genelyte, Susanne Kelfve

**Affiliations:** 1 Linkoping University, Norrkoping, Ostergotlands Lan, Sweden; 2 Jagiellonian University, Cracow, Malopolskie, Poland; 3 Linköping University, Norrköping, Sweden; 4 Linköping University, Norrköping, Ostergotlands Lan, Sweden

## Abstract

The labour market activity of older workers and their ability and disposition to maintain it depend on institutional conditions, age norms, labour demand and shifting overall economic conditions. The paper discusses exclusion and inequality in later working life from a European comparative perspective and emphasises shifts in late work and retirement patterns as well as later-life outcomes in Sweden and Poland. An emphasis is on changing institutional conditions on the national and branch level. Gendered risks for economic exclusion and later life precarity are stressed. Analyses for the two countries are contrasted with Germany and the UK. The analyses are part of the research program ‘Exclusion and Inequality in Late Working Life: Evidence for Policy Innovation Towards Inclusive Extended Work and Sustainable Working Conditions in Sweden and Europe – EIWO’ (2019-24). Analyses use data from SHARE and EU-SILC and address older workers of age 60 and older in Sweden, Poland, German and the UK. They find increasingly heterogeneous preretirement and transition patterns, new gender gaps and increasing risks of economic exclusion in retirement. Situations differ between countries with the prolongation of late working life in Sweden having a mostly positive effect on gender inequalities with low education and specific migrant groups as an exception. Poland is specific case due to unequally low retirement age for woman (60) and for men (65) with consequently large structural gender differences and increases in the process of increasing labour force participation of older workers and increasingly gendered risks for old-age economic exclusion.

